# The Regulation of Adaptation to Cold and Drought Stresses in *Poa crymophila Keng* Revealed by Integrative Transcriptomics and Metabolomics Analysis

**DOI:** 10.3389/fpls.2021.631117

**Published:** 2021-04-07

**Authors:** Yan Wang, Xin-Yu Li, Cai-Xia Li, Yuan He, Xin-Yi Hou, Xin-Rong Ma

**Affiliations:** ^1^Chengdu Institute of Biology, Innovation Academy for Seed Design, Chinese Academy of Sciences, Chengdu, China; ^2^University of Chinese Academy of Sciences, Beijing, China

**Keywords:** *Poa crymophila Keng*, cold and drought, transcriptome, widely targeted metabolomics, carbohydrates, flavone, phenylpropanoids

## Abstract

*Poa crymophila Keng* is highly adaptable to long-term low temperature and drought conditions, making it a desirable foraging grass of the Qinghai-Tibet Plateau. Here, the widely targeted metabolomics and comparative transcriptome analyses were utilized for the discovery of metabolites and genes in *P. crymophila* in response to cold and drought stresses. *P. crymophila* were exposed to −5°C for 24 h and recovered to 22°C for 48 h, as well as drought for 10 days followed by re-watering for 1 day. In total, 779 metabolic features were assigned to metabolites and 167,845 unigenes were generated. Seventeen compounds showed significant up-regulation (variable importance in project >1) under both stresses in the metabolic profiling, mainly annotated as carbohydrates, flavones, and phenylpropanoids. The genes which were positively correlated with these metabolites were assigned to pathways (sucrose-starch, raffinose, phenylpropanoid, and flavone metabolism) using the Mapman software package. Alpha-amylase, beta-fructofuranosidase, and sugar transport genes degraded the glucose and starch to small molecule sugars for the purpose of osmotic adjustment and to provide more energy for the growth of *P. crymophila* in an adverse environment. The induction of cinnamoyl-CoA reductase (CCR) and the MYB gene as well as the sharp increase in schizandrin, a kind of lignan, showed that this likely has the closest connection with the tolerance to both stresses. Four significantly induced flavone compounds are probably involved in reducing oxidative damage. Our results indicated that activation of the phenlypropanoid pathway plays the primary role in *P. crymophila* adapting to harsh environments. This study showed the mechanism of *P. crymophila* responding to both cold and drought stresses and showed the discovery of a new biological regulator against stresses.

## Introduction

*Poa* is an excellent gramineous forage grass and is widely distributed in Asia, Europe, and North America. *Poa crymophila Keng* grows in the meadows of hillsides and shrubs, and in the wetlands of open forests and riverbanks between 2,500 and 5,000 meters above sea level. *Poa crymophila Keng* cv. Qinghai is the primary grass species in mountain meadows of the Qinghai-Tibet plateau. It not only protects the local ecological environment, but also supports the development of animal husbandry. The long-lasting cold and drought in this region means that the grass has had to evolve to adapt to the adverse environment. To better understand the stress tolerances of this grass, we analyzed its transcriptome and metabolome in response to cold and drought.

When plants are subjected to abiotic stress, including cold and drought, many molecular and physiological processes are reconfigured. Numerous protective proteins and secondary metabolites are biosynthesized to help plants to adapt to these environmental stresses. Protective proteins, such as cold-regulated genes (*COR*), heat shock proteins (HSPs), and late embryogenesis abundant proteins (LEA), function as stabilizers of cell structures, chaperones and protectors of proteins, or antidotes to metal ions (Heidarvand and Amir, 2010; Bhargava and Sawant, 2013; Miura and Furumoto, 2013). Specialized metabolites, including carbohydrates, flavonoids, vitamins, phenylpropanoids, steroids, and anthocyanins are also induced by various abiotic stresses to act as osmoregulators and antioxidants (Zhu et al., 2007; Bhargava and Sawant, 2013; Brunetti et al., 2013). However, the substances which play key protective roles differ depending on the plant species and the stress.

At low temperatures, synthesis and accumulation of saccharides has been proven to be crucial to the improvement of cold tolerance in many plants (Kaplan and Guy, 2004; Rekarte-Cowie et al., 2008). Saccharides, such as fructose, mannose, pentose, and sugar, can protect cell structures by stabilizing membrane integrity and maintaining turgor and osmotic balance (Yano et al., 2005; Conde et al., 2011; Morkunas and Ratajczak, 2014). On the other hand, carbohydrates may directly quench reactive oxygen species (ROS) to mitigate oxidative damage under stress. The physical state of the plasma membrane also plays an important role in the cold adaptation of plants. Increasing unsaturated phospholipids and unsaturated fatty acids (FAs) and decreasing the proportion of cerebrosides can improve cold tolerance in a wide range of plants (Takahashi et al., 2013). Moreover, cold activates the synthesis of other secondary metabolites. For example, *Arabidopsis* rosettes placed in conditions with progressively decreasing temperatures in the non-freezing range showed an increase in expression of genes related to sucrose, proline, raffinose, tocopherol, and polyamine synthesis as well as phenylpropanoid and flavonoid metabolism (Usadel et al., 2008). In cold-treated tartary buckwheat (*Fagopyrum tataricum*), most of the phenylpropanoid biosynthetic transcripts are upregulated, and some organic acids derived from the tricarboxylic acid cycle increase (Jeon et al., 2018).

Similarly, plants coping with drought stress manipulate many physiological processes, involving many metabolites. Carbohydrates that contribute to growth under normal conditions may be used to synthesize solutes for osmotic adjustment under drought (Bhargava and Sawant, 2013). Drought stress enhances carbohydrate accumulation in star fruit (*Averrhoa carambola*), Kentucky bluegrass, cotton (*Gossypium hirsutum* L.), and *Phaseolus vulgaris* (Yang et al., 2013; Andrade et al., 2016; Wu et al., 2017; Zahoor et al., 2017). In Kentucky bluegrass, increased sucrose accumulation has been shown to be associated with superior turf performance during drought stress, whereas fructan accumulation contributes to rapid re-growth on re-watering (Yang et al., 2013). In cotton (*Gossypium hirsutum* L.), drought stress decreases starch content but increases sucrose content (Zahoor et al., 2017). Other secondary metabolites are also invoked to deal with drought stress. In *Saposhnikovia divaricata* subjected to progressive drought stress, aromatic alcohols and sesquiterpenes were identified to predominate in a total of 18 volatile components (Men et al., 2018). LC-MS analysis of leaf metabolites from 100 barley recombinant inbred lines has revealed that many metabolites, such as phenolic, terpenoid compounds, sinapic acid derivatives, acylated glycosides of flavones, and polyamine derivatives, are related to drought (Piasecka et al., 2017). Two edible fern (*Matteuccia struthiopteris*) species showed stronger resistance in the early stages of drought, due to increases in flavonoids, total phenols, and proanthocyanidins (Wang et al., 2019).

Many metabolites and their encoding genes, such as anthocyanins, sugar, phenylpropanoid, and some flavonols, are induced by both drought and cold stress (Shinozaki et al., 2003; Mierziak et al., 2014; Nakabayashi and Saito, 2015; Barrero-Gil et al., 2016; Pommerrenig et al., 2018), suggesting the existence of crosstalk between the drought and cold responding pathways. When antioxidant enzymes are either inactivated or insufficient during stress conditions, these metabolites probably play a vital antioxidant role in protecting plants from damage to DNA, proteins, and membrane lipids (Bartwal et al., 2013; Amelia et al., 2018). Yet, there remain important gaps in understandings of the molecular and physiological mechanisms underlying these adaptive processes, especially in non-model plants.

Hence, the goal of our study was to reveal how *Poa crymophila Keng* coped with low temperature and drought environments. We generated transcriptomes and metabolomes of *Poa* in response to cold stress and recovery temperature, as well as drought stress and re-watering. Bioinformatic analyses were performed to identify the major metabolites, regulation pathways, and candidate genes responding to both stresses. Consistencies and differences between the two stresses were also explored in terms of metabolites and molecules. The results provide insights into the molecular mechanisms behind the cold and drought tolerances of *P. crymophila* by exploring stress-tolerance metabolites and associated genes. These insights could be leveraged to develop new biological regulators against stresses or new grass varieties that have improved tolerance.

## Materials and Methods

### Plant Materials and Stress Treatments

*Poa crymophila Keng* cv. Qinghai seeds were planted in plastic pots (14 cm diameter, 25 cm length) filled with organic loam in October 2017 and were grown in a greenhouse at 18–25°C for 2 months in Chengdu (30.67°N, 104.06°E), Sichuan Province, China. There were at least 1,000 plants in each pot. Plants were watered by hand every 2 days. Prior to the experiment, plants were transferred to a growth chamber set to a temperature and light cycle of 22/16°C (14 h day/10 h night), at a relative humidity of 60% and an irradiance of 200 mmol·m^−2^s^−1^ (LI-6400/XT photometer, Li-Cor Inc., Lincoln, NE, USA) for 2 weeks. Four pots of plants acted as a control under normal conditions. To induce cold stress, four pots of plants were directly transferred to another growth chamber set at −5°C for 24 h (cold stress) with the same humidity and light conditions as above. the cold stress treated plants were then returned back to the control conditions for recovery from cold stress for 48 h. Leaves were randomly selected before treatment as a control (CK), 24 h cold stress, and 48 h recovery from cold (ReCold). Meanwhile, another four pots of plants were not watered for 10 days (Drought) in the third growth chamber, with otherwise normal conditions as per the control. They were re-watered and sampled after 48 h (ReDrought). At every sampling point, we collected five bunches of grass as biological replicates to conduct follow-up experiments, with at least 50 plants in each bunch.

### Illumina Deep Sequencing, *de novo* Assembly, and Functional Annotation

Total RNA samples were prepared using the Trizol^TM^ reagent (Invitrogen, Carlsbad, CA, USA), and subsequently purified with a cDNA library constructed using the Truseq™ RNA Sample Prep Kit (Illumina, San Diego, CA, USA) following the manufacturer's instructions. After quality control using an Agilent 2100 Bioanaylzer and the ABI StepOnePlus Real-Time PCR System, the cDNA libraries were sequenced on Illumina HiSeq™ 4000 (Illumina) at BGI (Shenzhen, China). Each sample yielded more than 5 Gb data. All RNA-Seq reads were deposited to the Sequence Read Archive database (http://www.ncbi.nlm.nih.gov/Traces/sra/) under accession number SRX2725266.

The clean reads, which were obtained by filtering raw reads from sequencing machines using the internal filter_fq software of BGI (Shenzhen, China), were used for bioinformatics analysis. *De novo* assembly of the *P. crymophila* transcriptome was conducted using Trinity (release 20130225) (http://trinityrnaseq.sourceforge.net/) under default parameters (Grabherr et al., 2011). The quality of the assembly was determined using total length, mean length, N50 number, and the length distribution of contigs and unigenes. The assembled unigene sequences were aligned to the following protein databases: NR (release 20130408), Swiss-Prot (release 2013_03), the Kyoto Encyclopedia of Genes and Genomes (KEGG, release 63.0), Cluster of Orthologous Groups of proteins (COG) (release 20090331) by blastx (e-value <0.00001); and nucleotide database NT (release 20130408) by blastn (e-value <0.00001) (http://blast.ncbi.nlm.nih.gov/Blast.cgi). These unigenes were annotated for their function through identifying proteins with the highest sequence similarities. GO annotation of unigenes was conducted with NR annotation using the Blast2GO program (release 2012-08-01) (https://www.blast2go.com/) (Conesa et al., 2005).

### Differentially Expressed Genes (DEGs)

Unigene expression levels were calculated in terms of the fragments per kilobase of exon model per million (FPKM) using the software package RSEM (RNA-Seq by Expectation Maximization) (Li and Dewey, 2011). Based on the FPKM of unigenes, the Noiseq package was used to calculate expression differences between the treatment groups (Tarazona et al., 2011). The DEGs were screened according to a threshold of |log2FC| ≥1 and probability ≥0.8.

### Metabolite Analyses

Three biological replicates (−1, −2, −3) in every sampling point were used to detect metabolite features and study the metabolome in widely targeted analysis at Wuhan Metware Biotechnology Co. LTD (Wuhan, China). Frozen powder plant leaves (100 mg) were extracted overnight at 4°C with 1.0 ml 70% aqueous methanol. To improve the extraction efficiency, the samples were shaken by a vortex three times during this process. Samples were then centrifuged at 4°C, 10,000 × g for 10 min. The extracts were absorbed (CNWBOND Carbon-GCB SPE Cartridge, 250 mg, 3 ml; ANPEL, Shanghai, China, www.anpel.com.cn/cnw) and filtrated (SCAA-104, 0.22 μm pore size; ANPEL, Shanghai, China, http://www.anpel.com.cn/) to perform LC-MS analysis on an UPLC-ESI-MS/MS system (UPLC, Shim-pack UFLC SHIMADZU CBM30A system, www.shimadzu.com.cn/; MS, Applied Biosystems 6500 Q TRAP, www.appliedbiosystems.com.cn/) (Chen et al., 2013; Dunn et al., 2013). Dichloro-phenylalanine was added to each sample before analysis as the internal standard to check the reliability and stability of the compounds. Total ions current (TIC) of quality control (QC) samples and multimodal maps of detected metabolites via multiple reaction monitoring (MRM) model were acquired on a triple quadrupole linear ion trap mass spectrometer (Chen et al., 2013), equipped with an ESI (electrospray ionization) Turbo Ion-Spray interface, operating in a positive ion mode and controlled by Analyst 1.6.3 software (AB Sciex). QC was operated by QC samples which are prepared from a mixture of sample extracts. One QC sample is added every 10 test samples to monitor the reproducibility of samples under the same treatment method. The overlapping TIC diagrams of different QC samples displayed the high repeatability of metabolite extraction and detection.

The metabolites were qualitatively and quantitatively determined according to secondary spectral information after removing isotopic signals, repeated signals containing K^+^ ions, Na^+^ ions, NH^4+^ ions, and fragment ions from larger molecular weight substances. The multimodal maps of metabolite detection via MRM showed the substances that can be detected in the samples, with each different color of the mass spectrum peak representing one detected metabolite. The qualitative analysis was based on RT and fragment ion under the given ratio of declustering potential to collision energy. If the generated fragment ion can be identified with corresponding standard fragment from an authentic chemical standard (BioBioPha Co., LTD, Kunming, China and Sigma-Aldrich, Merck KGaA, Darmstadt, Germany), the fragment was considered as definitive identification. If the fragment cannot be identified with standard fragments, the size of the fragment was used to speculate the chemical groups in order to reconstruct the structure of matter and putatively annotate compounds with the self-built database MWDB (Metware Database, Wuhan Metware Biotechnology Co. LTD, Wuhan, China) (Dunn et al., 2013). The quantitative analysis of metabolites was based on the relative content which was represented by the integration of the peaks area of each chromatographic peak using Multiaquant software (Fraga et al., 2010). Because of the content differences of every detected metabolite between different samples, the mass spectral peaks of all detected metabolites were corrected according to the retention time and peak type of every metabolite in different samples to ensure the accuracy of the qualitative and quantitative analysis.

### Analysis, KEGG Annotation, and Enrichment of Differentially Expressed Metabolites (DEMs)

Partial Least Squares-Discriminant Analysis (PLS-DA) can maximize the differentiation between groups and is beneficial when searching for different metabolites. Accordingly, PLS-DA was used to calculate the correlations between the different groups of metabolome data (Thévenot et al., 2015). The differential metabolites between different groups can be preliminarily screened out on the basis of variable importance in project (VIP) which was obtained from the PLS-DA. The fold change was calculated by dividing the mean value of the signal peak area of the detected substance between different groups, in consideration of biological duplication in the *Poa* metabolome. When the fold change exceeded 2 or was <0.5, and VIP >1, the difference was considered significant.

Differential metabolites were then mapped to the KEGG database (Kanehisa and Goto, 2000) to carry out enrichment analysis. The Rich factor is the ratio of the number of DEMs in a certain pathway to the total number of metabolites detected and annotated in the corresponding pathway.

### Differential Correlation Analysis and Correlation Network Diagram

Correlation analysis was conducted for DEMs and genes using the COR program in R (Chong and Xia, 2018). The positively related genes with Pearson's correlation coefficients (PCC) >0.9 were selected and further filtered on the basis of gene length exceeding 750 bp.

The selected genes were then analyzed using the MapMan software package (http://mapman.gabipd.org/web/guest/home), which includes two freely available programs (Schwacke et al., 2019). First, each input gene was given a function annotation item “Bin” based on the reference database in MapMan. The pathway interpretation of these genes was then visualized in MapMan. After that, network visualization between the target metabolites and genes was completed using the Cytoscape software package (Shannon et al., 2003).

### Quantitative Real-Time (qRT) -PCR Verification

The purified RNA samples were reverse-transcribed using the PrimeScript RT Reagent Kit with gDNA Eraser (Takara, Dalian, China) following the manufacturer's protocol. In the 4,286 upregulated genes with a length over 750 bp, 16 unigenes were selected for the qRT-PCR assay. Gene specific qRT-PCR primers (18–22 bp) were designed using Premier 5.0 software (Premier Biosoft International, Palo Alto, CA). qRT-PCR was performed using TB Green® Premix Ex Taq™ II (Tli RNaseH Plus) (Takara Bio Inc., Shiga, Japan) in an ABI Quantstudio 3 Real-Time PCR System (Applied Biosystems, Foster City, CA, USA). PCR conditions were 30 s at 95°C, followed by 40 cycles of heating at 95°C for 5 s and annealing at 60°C for 34 s. Three replicates were performed, and the amplification specificity were checked by melting curves. The relative expression level of each gene, namely the fold change (FC) of gene expression between treated samples and the control sample, was calculated using 2^−ΔΔCt^, and the beta-actin gene from the Poa transcriptome served as the reference gene.

## Results

### Transcriptomes of *Poa crymophila Keng* cv. Qinghai

**Figure 1:**
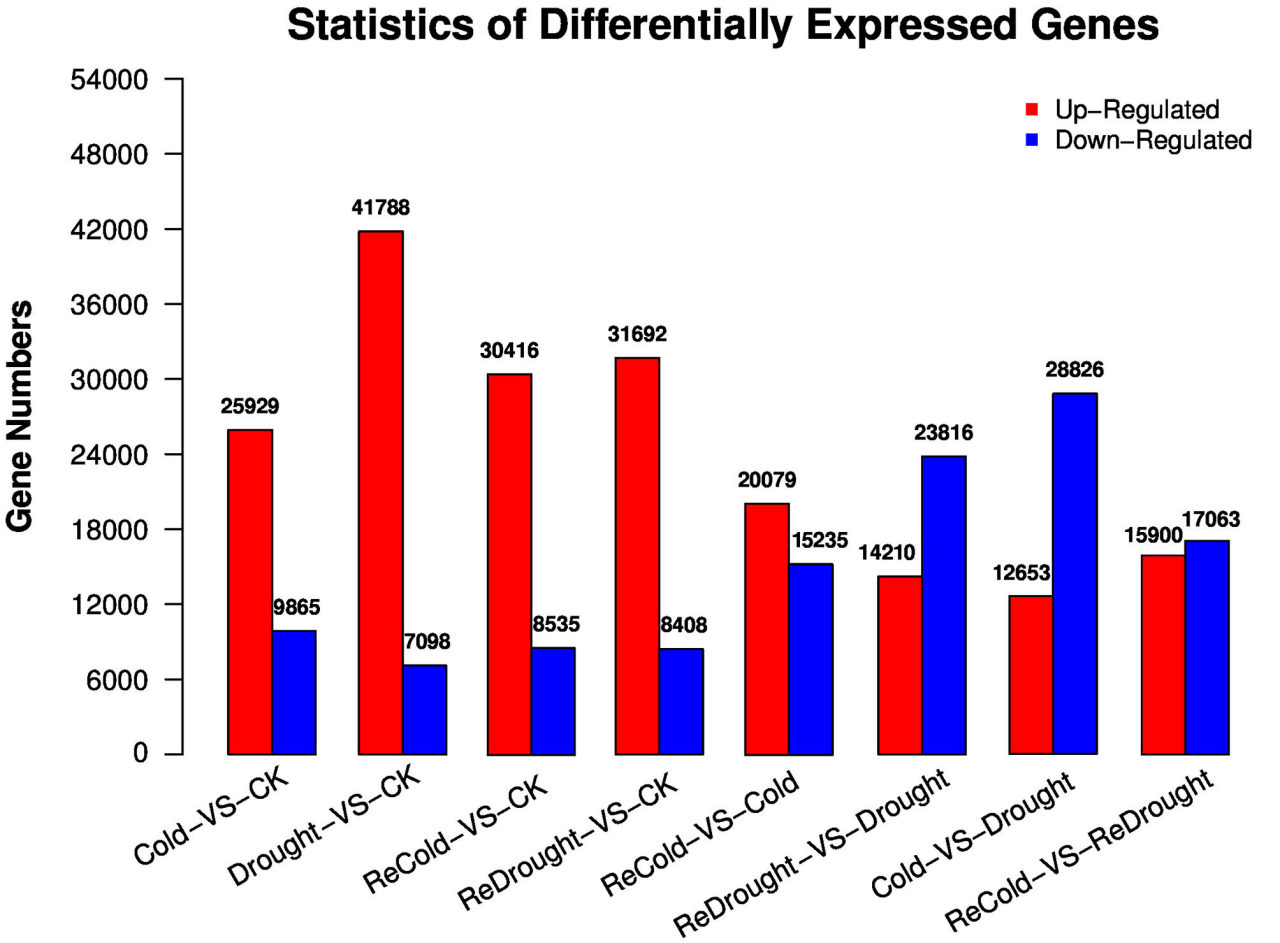
Significantly differentially expressed genes (DEGs) in *Poa crymophila Keng* cv. Qinghai transcriptomes in response to cold and drought stress and recovery from the two stresses.

In transcriptomes of *P. crymophila* in response to cold, drought, and recovery from both of these stresses, 167,845 unigenes were detected (Supplementary Table 1). The total length of the unigenes was 103,424,584 nt, the average length was 616 nt, and N50 was 804 nt. The transcriptome was functionally annotated to NR, NT, Swiss-Prot, KEGG, COG, GO, PFAM, and InterPro databases, resulting in the annotation of 84,341, 102,333, 45,820, 52,310, 30,737, 45,724, 35,232, and 42,881 unigenes, respectively. In total, 112,353 unigenes were annotated across the different databases, reaching 66.94% of total unigenes (Supplementary Table 2). Next, we screened DEGs between different experimental conditions based on FPKM values. The results revealed that cold stress significantly upregulated 25,929 unigenes and down-regulated 9,865 unigenes. Drought stress significantly upregulated 41,788 unigenes and down-regulated 7,098 unigenes. Compared with the control group, the plants recovering from cold stress contained 30,416 upregulated DEGs and 8,535 down-regulated DEGs. The re-watered plants, after drought treatment, contained 31,692 upregulated DEGs and 8,408 down-regulated DEGs. Compared with the treatment group, recovering from cold stress upregulated 20,079 DEGs and down-regulated 15,235 DEGs, whilst re-watering upregulated 14,210 DEGs and down-regulated 23,816 DEGs (Supplementary Table 1, Figure 1).

Under cold stress, the pathways in which DEGs significantly enriched mainly included plant-pathogen interactions, plant hormone signal transductions, phenylpropanoid biosynthesis, and starch and sucrose metabolism. Under drought stress, the pathways in which DEGs significantly enriched are FA metabolism, biosynthesis and degradation, other glycan degradation, nitrogen metabolism, selenocompound metabolism, and plant-pathogen interaction. Many regulated genes and function genes were induced to respond to cold and drought stresses, such as transcription factors, protein kinases, CORs, HSPs, LEAs, oxidation-reduction enzymes, and some unidentified proteins. It is not straightforward to determine which genes play key roles in *P. crymophila* in response to the two abiotic stresses. In this study, we first searched for the key metabolites which accumulated in *P. crymophila* suffering from the cold and drought stresses, which was the most direct evidence of the adaptability of *Poa*. Next, the corresponding pathways and core genes of these metabolites were traced in the transcriptome.

### Metabolome of *Poa crymophila Keng* cv. Qinghai

In the metabolic profiling of *P. crymophila*, 779 metabolite features were identified and annotated as metabolites by widely targeted analysis. They were classified into carbohydrates, lipids, flavones, phenylpropanoids, alcohols, alkaloids, terpene, organic acids and derivatives, amino acids and derivatives, and vitamins and derivatives (Supplementary Table 3). Compared with the control group, the groups of cold, drought, recovery from cold, and re-watering after drought resulted in 214, 203, 223, and 105 significantly DEMs, respectively. However, compared with the treatment group, 128 DEMs were detected in the plant group recovering from cold, and 160 DEMs were detected in the re-watering group after drought. The number of upregulated and down-regulated DEM_S_ are listed in Table 1.

**Table 1:** Significantly differentially expressed metabolites (DEMs) in *Poa crymophila Keng* cv. Qinghai in response to cold and drought stress and recovery from the two stresses.

**Group name**	**All DEMs**	**Down regulated DEMs**	**Up regulated DEMs**
CK-M_vs._Cold-M	214	111	103
CK-M_vs._Drought-M	203	141	62
CK-M_vs._ReCold-M	223	93	130
CK-M_vs._ReDrought-M	105	64	41
Cold-M_vs._ReCold-M	128	32	96
Drought-M_vs._ReDrought-M	160	42	118

In the process from cold stress to recovery, carbohydrates firstly increased and then decreased, and the content of lipids, amino acids and derivatives, nucleotides and derivatives, and alcohols continuously increased. Flavones showed a downward trend in the process (Table 2). For drought stress, carbohydrates and amino acids, and derivatives increased in concentration while other types of metabolites decreased. However, most types of metabolites increased in content after re-watering, except for carbohydrates and amino acids (Table 2).

**Table 2:** The abundance changes of metabolite types from stress to recovery in response to cold and drought.

	**Cold/CK**			**Re-cold/cold**			**Drought/CK**			**Re-drought/drought**		
	**Sum**	**Up**	**Down**	**Sum**	**Up**	**Down**	**Sum**	**Up**	**Down**	**Sum**	**Up**	**Down**
Lipids	31	31	0	14	14	0	19	3	16	22	18	4
Sterides	1	1	0	1	1	0						
Organic acids and derivatives	22	6	16	10	9	1	17	5	12	13	12	1
Indole derivatives	1	1	0	1	1	0	2	2	0			
Isoflavone	4	0	4				7	0	7	6	6	0
Vitamins and derivatives	5	3	2	2	1	1	5	1	4	3	3	0
Terpene	2	1	1	2	2	0	1	1	0	1	0	1
Carbohydrates	6	6	0	5	0	5	11	10	1	5	0	5
Alkaloids	11	5	6	5	4	1	8	1	7	7	6	1
Others	13	6	7	5	4	1	6	3	3	4	2	2
Flavanone	3	2	1	2	1	1	7	2	5	4	4	0
Flavonoid	9	1	8	1	1	0	10	2	8	7	6	1
Flavonol	11	1	10	4	0	4	11	0	11	7	7	0
Flavone	34	10	24	18	7	11	55	15	40	35	26	9
Anthocyanins	1	0	1				3	0	3	2	2	0
Nucleotide and derivates	12	9	3	21	20	1	5	1	4	11	10	1
Phenolamides	10	2	8	13	12	1	5	3	2	5	5	0
Polyphenol	2	1	1	2	1	1	1	0	1	1	1	0
Alcohols	3	3	0	1	1	0	4	3	0	2	1	1
Phenylpropanoids	22	6	16	7	5	2	15	4	11	7	5	2
Amino acid and derivatives	11	8	3	10	9	1	10	6	4	17	3	14
Proanthocyanidins							1	0	1	1	1	0

### The Concentration of Some Metabolite Significantly Increasing in Response to Both Cold and Drought Stresses

**Figure 2:**
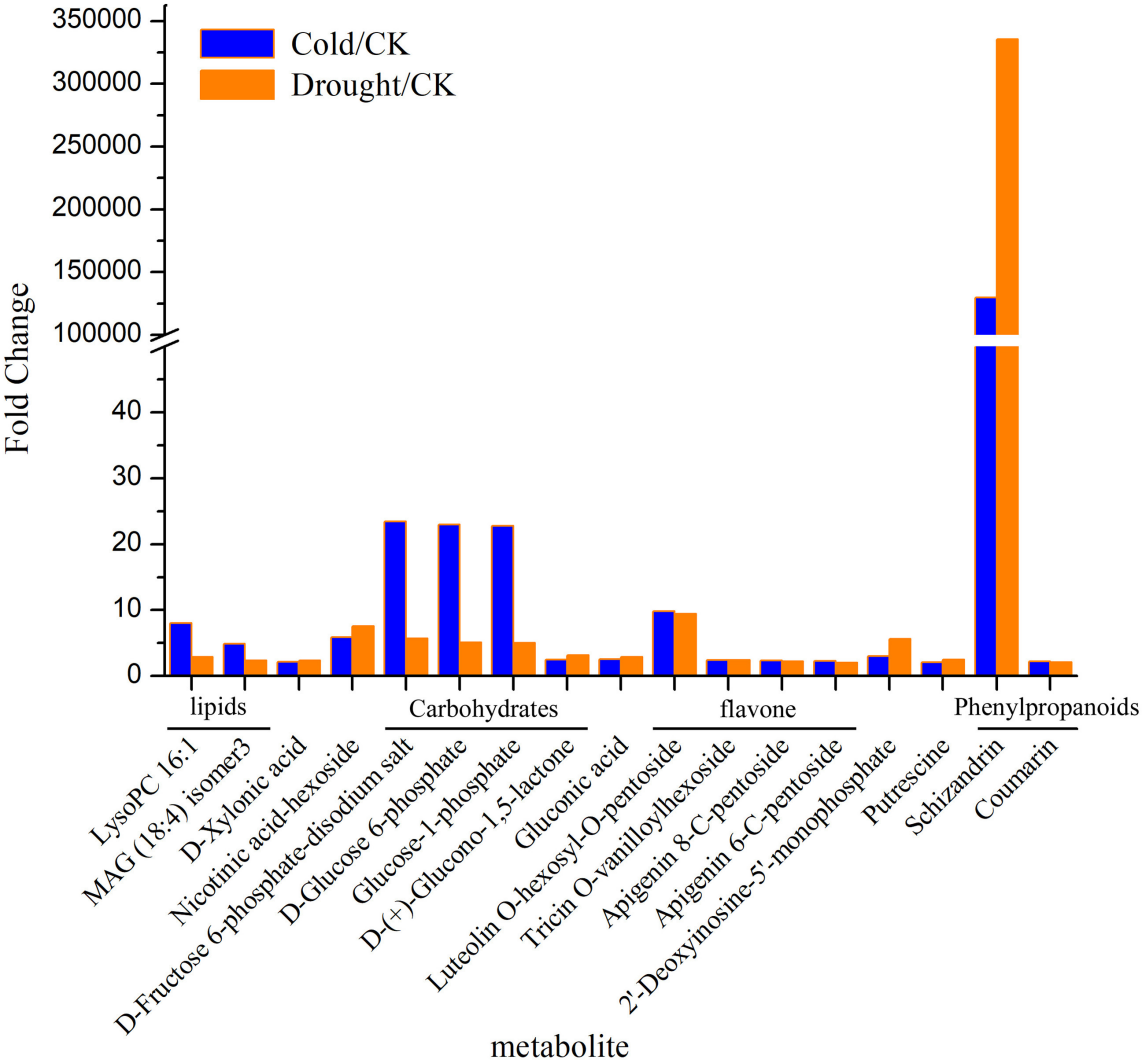
The significantly up-regulated metabolites under both cold and drought stresses.

Metabolites which responded to both cold and drought were selected by Venn diagram analysis. On this basis there were 17 significantly upregulated metabolites under both stresses (Figure 2, Supplementary Table 3). Ten of them were confidently identified with an authentic chemical standard (D-(+)-Glucono-1,5-lactone, Gluconic acid, Putrescine, D-Glucose 6-phosphate, Coumarin, D-Xylonic acid, Schizandrin, Glucose-1-phosphate, D-Fructose 6-phosphate-disodium salt and LysoPC 16:1), and the others (Nicotinic acid-hexoside, Tricin O-vanilloylhexoside, MAG (18:4) isomer3, O-hexosyl-O-pentoside, Luteolin, Apigenin 6-C-pentoside, Apigenin 8-C-pentoside, and 2′-Deoxyinosine-5′-monophosphate) were putatively annotated by comparing the mass spectrum to data collected in spectral libraries (Figure 2). Therein, schizandrin (pmf0166) showed the most significant increase in expression abundance (over 100,000 times). Luteolin (pmb0566) and nicotinin acid-hexoside (pma1751) presented more than five times the enhancement in expression abundance. Moreover, three metabolites belonging to carbohydrates, D-Fructose 6-phosphate-disodium salt (pmf0220), D-Glucose 6-phosphate (pme3160), and Glucose-1-phosphate (pmf0035), were also induced to express over 22 times by cold stress and above five times by drought. In addition, the abundance of LysoPC 16:1 (pmb0165) was more increased by cold than drought.

### Pathway Analysis of Upregulated Genes (>750 bp) Correlated With Candidate Metabolites

A correlation analysis between the metabolites and genes indicated that 19,106 genes were positively related to the 17 metabolites with Pearson's correlation coefficients >0.9 (Supplementary Table 4). Among them, 14,657 upregulated genes are linked with schizandrin (pmf0166). Considering the further experiment concerning gene function, genes with a length <750 bp were omitted, leaving 4,286 upregulated genes with a length over 750 bp for analysis of metabolic pathways using MCC and Mapman software. The results showed that 64 genes were mapped in secondary metabolism and 37 in primary metabolism (Supplementary Table 4).

**Figure 3:**
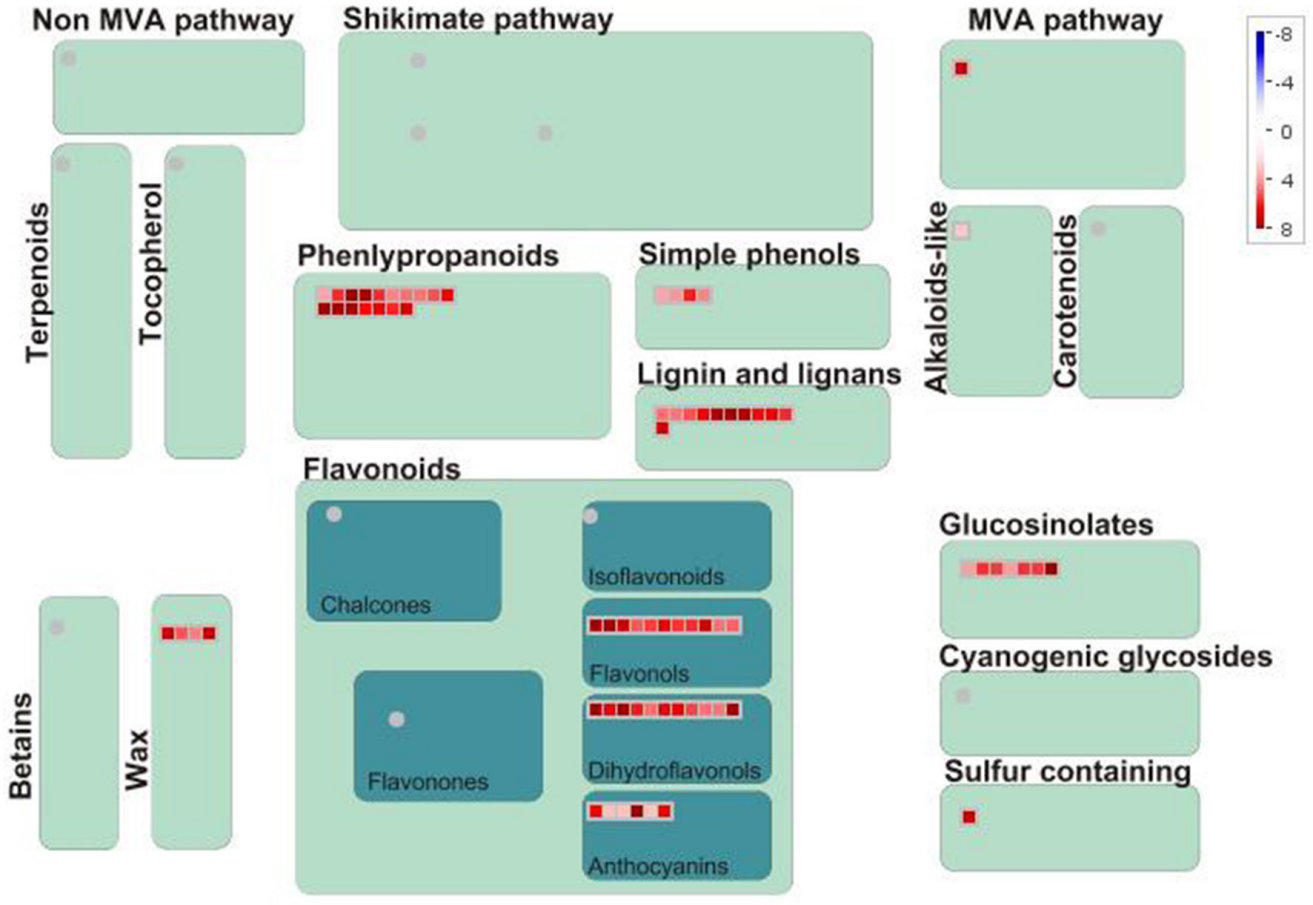
The correlated genes which positively responded to cold and drought were mapped to secondary metabolism.

**Figure 4:**
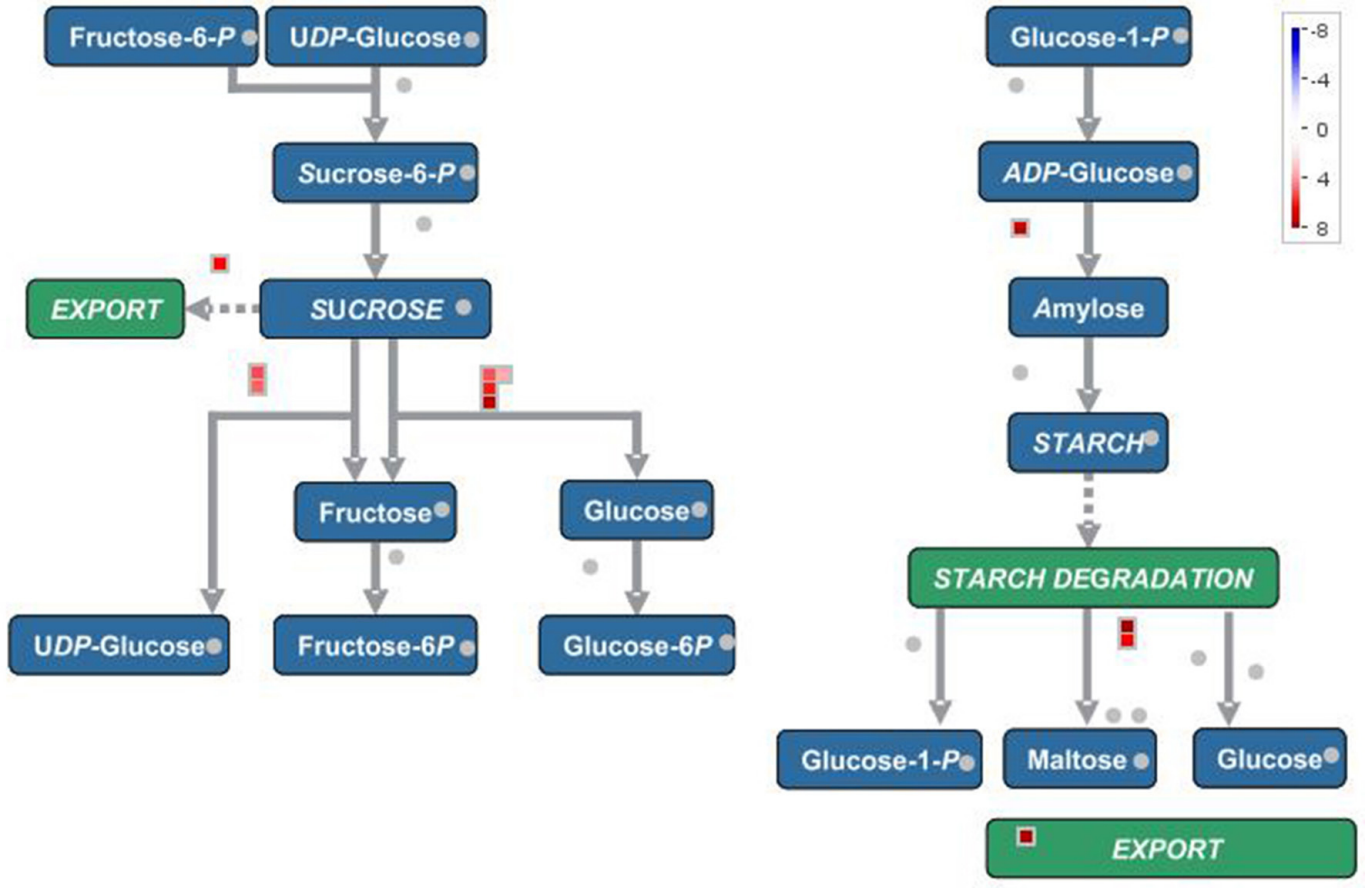
The correlated genes which positively responded to cold and drought were mapped to sucrose-starch pathway in primary metabolism.

In terms of secondary metabolism, genes mainly focused on the following pathways: phenlypropanoid, flavonol, anthocyanin, as well as lignin and lignan (Figure 3). In primary metabolism, genes were primarily mapped to the sucrose-starch and raffinose metabolism pathways (Figure 4), and some genes were connected to lipid biosynthesis, photosynthesis, and plant glycolysis.

**Figure 5:**
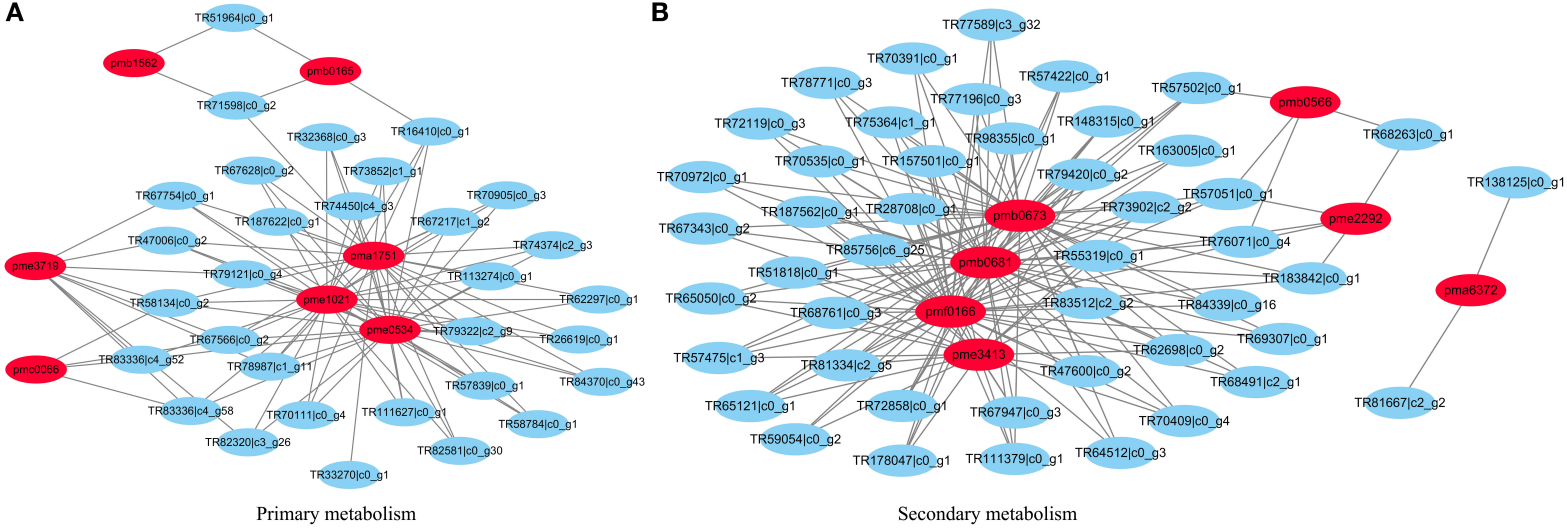
The established networks between metabolites and their correlated genes. **(A)** The network graph of primary metabolism. **(B)** The network graph of secondary metabolism.

Moreover, a network was established between different metabolites and their corrected genes. In the network graph of secondary metabolism, pmb0637, pmb0681, pmf166, and pme3413 coalesced, and most genes were linked to these four metabolites. While pmb0566, pme2292, and pma6372 were involved with respect to the remaining genes. For the primary metabolism network, pma1751, pme1021, and pme0534 were closer and related to more correlated genes than pme3719, pme0066, pmb0165, and pmb1562 (Figure 5).

### The Key Genes in *Poa crymophila Keng* Responding to Cold and Drought Stresses

In the correlated genes, which were mapped in Mapman, the DEGs with a probability ≥0.8 in both stresses were screened out as candidate genes involved in the tolerance of *Poa* to cold and drought stimulations (Table 3). In primary metabolism, seven genes responded to both stresses and were linked to pme0534, pma1751, and pme1021. Their functions involved glucose transportation and degradation, starch degradation, wax-ester synthase, and FA synthesis. In addition, raffinose synthase showed the highest expression levels in both stresses, and it was significantly induced by drought but not by cold. A similar trend was revealed with respect to 1-fructosyltransferase, but its expression level was lower than raffinose synthase. In secondary metabolism, eight genes responded to both stresses and their notation focused on cinnamoyl-CoA reductase and flavonol synthase. Moreover, one hydroxymethylglutaryl-CoA reductase (TR83164|c3_g4) exhibited a remarkable increase in expression level to 460.37 in response to the cold stress, but its abundance was not changed by drought. Finally, some genes annotated as asulfotransferase and UDP-glycosyltransferase were induced by drought stress.

**Table 3:** The candidate genes in response to both cold and drought stimulations in *Poa crymophila Keng* cv. Qinghai (Probability >0.8).

	**TR-id**	**log_**2**_(Cold/CK)**	**Probability**	**log_**2**_(Drought/CK)**	**Probability**	**KEGG-Annot**
Secondary metabolism	TR57051|c0_g1	10.35	0.99	9.38	0.97	Rhamnosyltransferase
	TR57502|c0_g1	10.06	0.98	8.45	0.91	Flavonol synthase
	TR72858|c0_g1	9.54	0.97	11.10	0.99	Cinnamoyl-coa reductase
	TR68491|c2_g1	9.41	0.96	10.99	0.99	Myb proto-oncogene protein, plant
	TR72119|c0_g3	9.08	0.95	10.84	0.99	Cinnamoyl-coa reductase
	TR75364|c1_g1	6.66	0.92	7.172	0.92	2′-deoxymugineic-acid
	TR78771|c0_g3	8.60	0.90	8.61	0.91	Cinnamoyl-coa reductase
	TR64512|c0_g3	8.50	0.90	8.23	0.88	FLS1, FLS4; flavonol synthase
Primary metabolism	TR82581|c0_g30	8.61	0.90	10.90	0.99	Sugar porter family MFS transporter
	TR78987|c1_g11	8.01	0.85	9.92	0.98	Alpha-amylase
	TR79121|c0_g4	8.37	0.90	9.42	0.97	Solute carrier family 35
	TR70111|c0_g4	8.04	0.97	8.19	0.97	3-hydroxyacyl-dehydratase
	TR67217|c1_g2	10.35	0.99	8.78	0.94	Wax-ester synthase
	TR67628|c0_g2	7.77	0.85	8.28	0.91	Beta-fructofuranosidase
	TR47006|c0_g2	8.25	0.90	7.98	0.86	–

### qRT-PCR Validation of Differentially Expressed Unigenes From RNA-Seq

**Figure 6:**
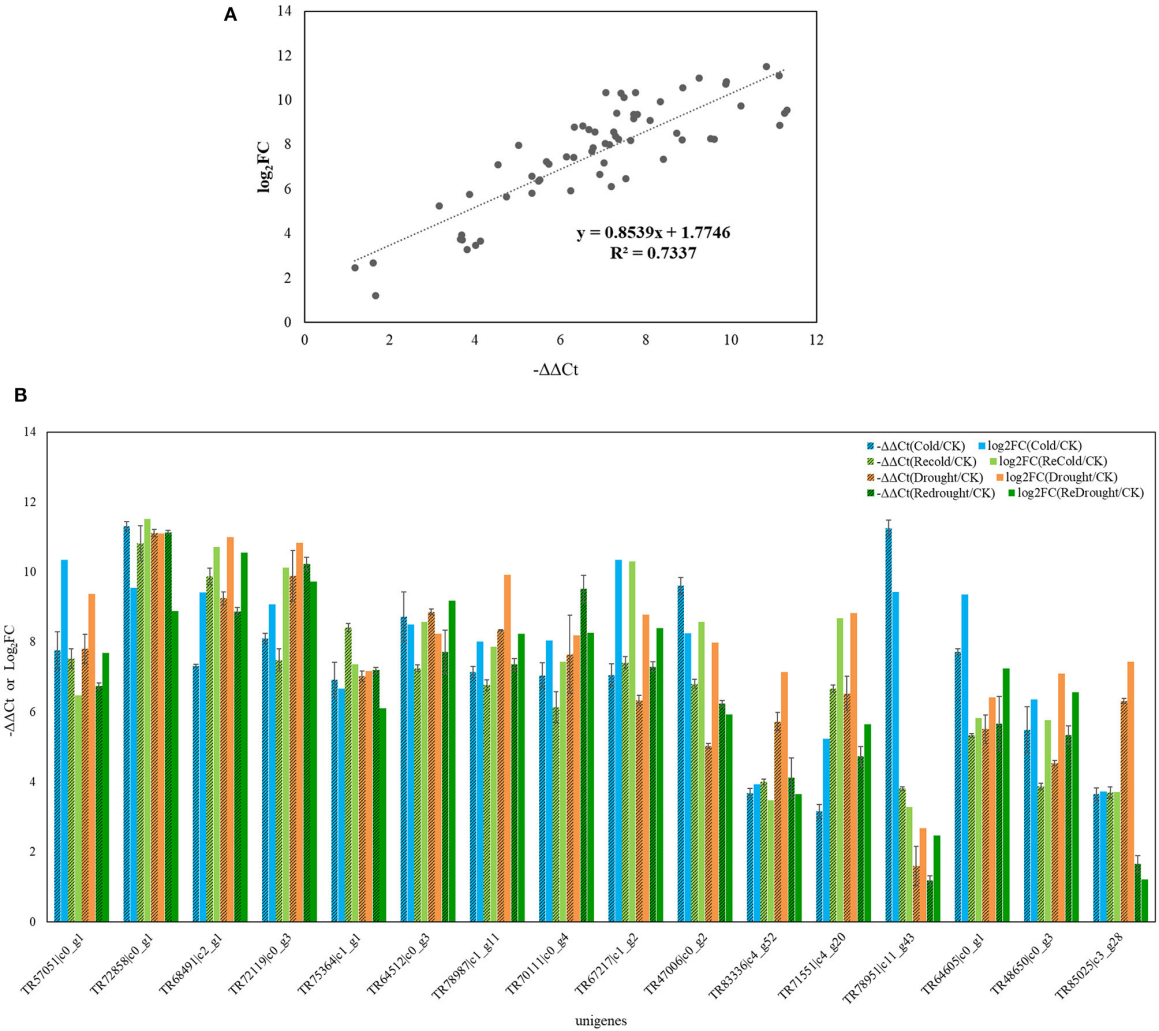
qRT-PCR verifying the accuracy of RNA-seq. Sixteen unigenes were selected for the qRT-PCR assay and results are from three biological replicates. Beta-actin gene from *Poa crymophila Keng* transcriptome was served as reference gene. The fold change (FC) of gene expression between treated samples and control sample was calculated using 2^−ΔΔCt^, and ${\text{Log}}_{2}^{\text{FC}}$ = –ΔΔCt. the ${\text{Log}}_{2}^{\text{FC}}$ of each unigene in qRT-PCR and transcriptome was compared to verify the stability and accuracy of the RNA sequencing. **(A)** The fitting line of ${\text{Log}}_{2}^{\text{FC}}$ of each unigene in qRT-PCR and transcriptome. **(B)** The expression change of each unigene in qRT-PCR (–ΔΔCt) and transcriptome (${\text{Log}}_{2}^{\text{FC}}$).

This study focused on genes that were positively related to the metabolites in response to two abiotic stresses, we therefore chose 16 unigenes from 4,286 upregulated genes with a length over 750 bp to perform the qRT-PCR analysis. Some of those genes were involved in phenlypropanoid, sucrose-starch pathways, and raffinose metabolism pathways. The qRT-PCR results are generally consistent with expression changes of these genes in the transcriptome (Figure 6), suggesting the reliability of the Illumina RNA-seq result.

## Discussion

### Analysis of the Overall Response to Cold and Drought in *Poa crymophila Keng*

*Poa crymophila Keng* grown on the Qinghai-Tibet plateau has evolved a high tolerance and is adapted to the cold and arid environment there. In this study, we sought to further explore the stress-tolerance of the grass via metabolome and transcriptome analyses. In the *P.crymophila* transcriptome, Many DEGs were identified and involved in almost all aspects of the metabolic process, which made identifying the genes that are most important for stress responses difficult. Metabolites directly function to help grass resist stresses. So, this study commenced by searching for the main metabolites under two stresses and then traced the associated pathways and genes. Here, we paid particular attention to the induced metabolites, because they should be positively linked to the acquirement of tolerances.

Carbohydrates first increased and then decreased in the process from cold and drought stresses to recovery. Carbohydrates accumulating in the cytoplasm can not only maintain turgor and osmotic balance, but also do not interfere with normal cellular metabolism (Chen and Murata, 2002; Conde et al., 2011). Moreover, we speculated that carbohydrates also provide more energy resource *to Poa* to synthesize particular anti-stress compounds for surviving under adverse conditions.

Under low temperature, lipids, amino acids, nucleotides of *P. crymophila* continuously increased in abundance from cold treatment to recovery. The promotion of unsaturated lipids may reduce the temperature at which the plasma membrane solidifies and thus improves its fluidity (Takahashi et al., 2013). The increased abundance of amino acids and nucleotides may contribute to protective proteins, including CORs, dehydrins, and LEAs. A considerable number of genes encoding these proteins have recently been identified in many plant species in response to cold stress (Miura and Furumoto, 2013). Under drought stress, only amino acids and their derivatives showed more up-regulation, which may connect to LEA because LEA genes have been reported to enhance the drought tolerance of transgenic maize, tobacco, and upland cotton (Magwanga et al., 2018; Minh et al., 2019).

Once the two stresses were withdrawn, growth of *Poa* rapidly resumed and most metabolites showed a concentration callback. This suggested that *Poa* can withstand repeated harm from freezing and long periods of drought.

### The Metabolites Induced by Both Cold and Drought Stresses

**Figure 7:**
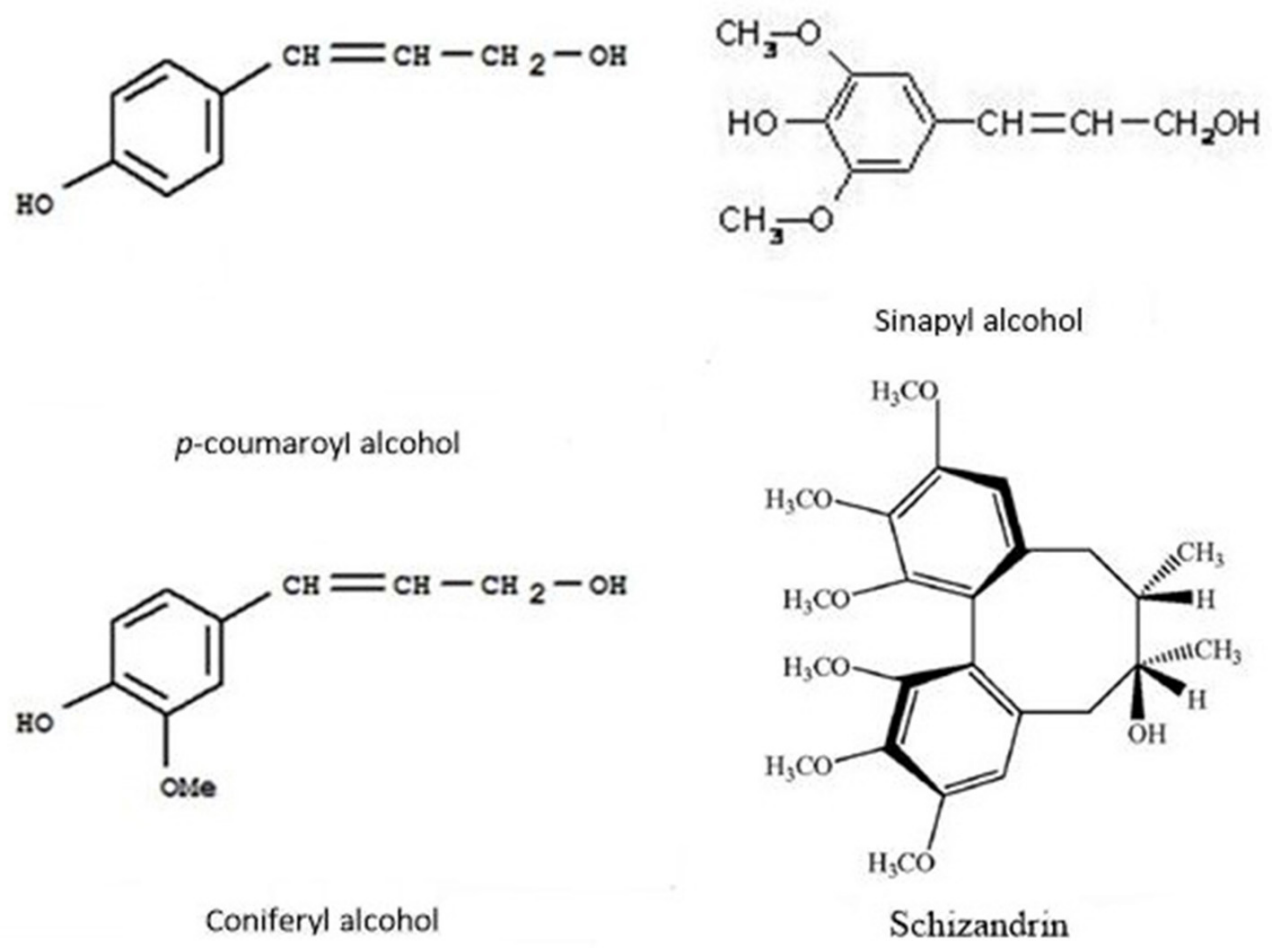
Molecular structures of p-coumaryl alcohol, coniferyl alcohol, sinapyl alcohol, and schizandrin.

In this study, 17 metabolites were significantly induced by both stresses. Among them, the abundance of schizandrin (pmf0166) increased over 100,000 times and was far higher than the other 16 metabolites (Figure 2). Schisandrin, a kind of lignan, has been studied as a plant-based medicine (Sowndhararajan et al., 2018) and has been shown to protect from neurotoxicity and enhance cognitive functions in the cell line and animal models (Egashira et al., 2008; Xu et al., 2012). However, there is a dearth of research in terms of its function in the plant itself. Both lignan and lignin are synthesized by monolignols via the phenylalanine pathway. Monolignols are divided into p-coumaryl alcohol, coniferyl alcohol, and sinapyl alcohol, resulting in the formation of H-hydroxyphenyl, G-guaiacyl, and S-syringyl lignin, respectively (Gray et al., 2012). Lignan is thought to come from G- and S- lignin. Schizandrin is a kind of dibenzocyclooctene lignan and three oxygen atoms are attached to every benzene ring via a C-O bond (Sowndhararajan et al., 2018). Comparing the molecular structures of schizandrin and the three monolignols, it was inferred that schizandrin is formed by sinapyl alcohol because there are also three C-O bonds in sinapyl alcohol (Figure 7). Lignin-like polymers have been referred to as stress-lignin or defense-lignin, and they can be induced by external biotic and abiotic stresses such as pathogen attacks, water deficits, high light, ozone, heavy metals, and mechanical stress (Gray et al., 2012). Schizandrin in *P. crymophila* should be regarded as a “stress-lignin” which warrants exploration in terms of its function and regulatory mechanism.

**Figure 8:**
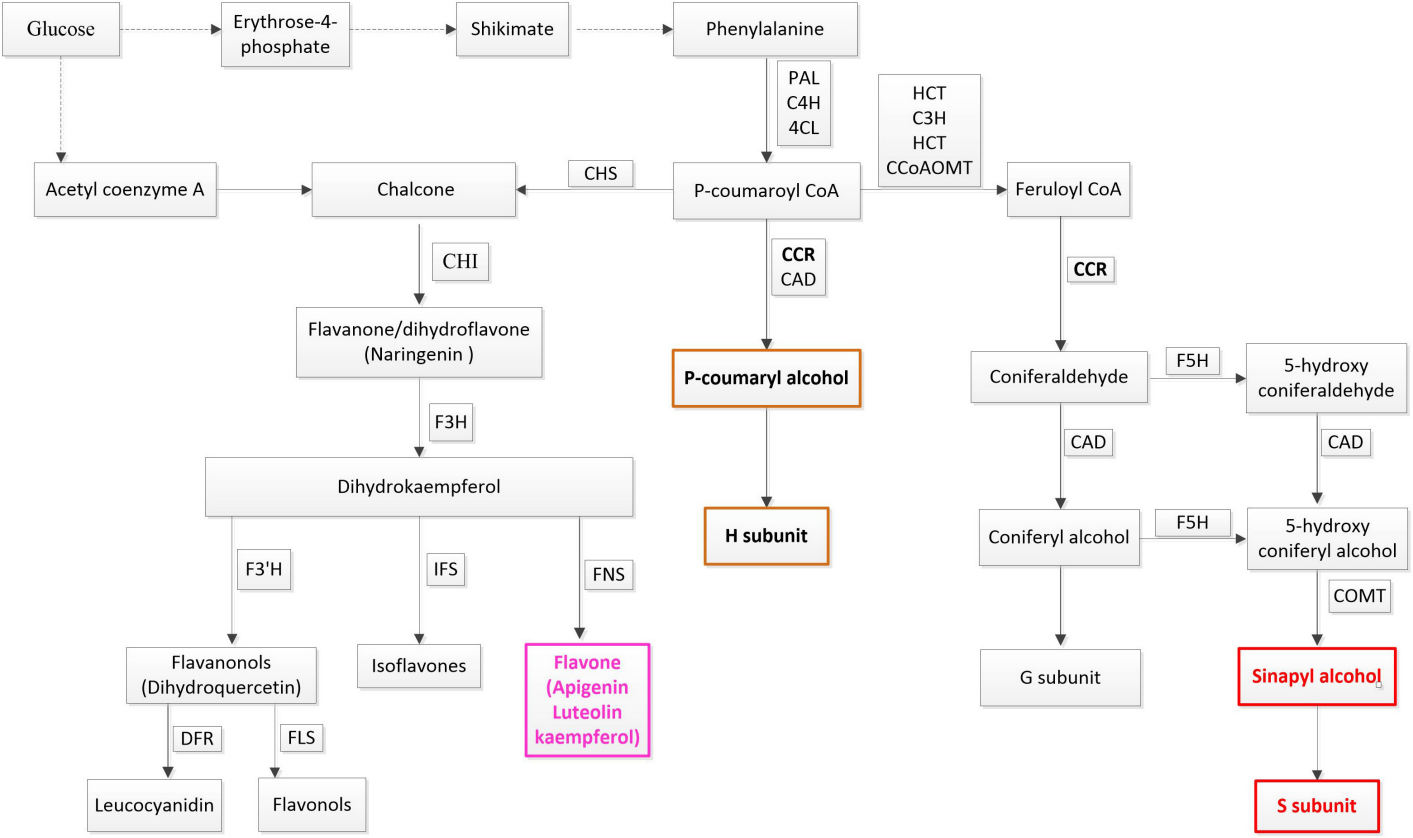
Phenylpropanoid pathway. PAL, phenylalanine ammonia-lyase; C4H, cinnamate 4-hydroxylase; 4CL, 4-coumarate-CoA ligase. The monolignol biosynthetic branch: CCR, cinnamoyl-CoA reductase; CAD, cinnamyl alcohol dehydrogenase; HCT, hydroxycinnamoyl-CoA shikimate/quinate hydroxycinnamoyl transferase; C3H, 4-coumarate 3-hydroxylase; COMT, caffeic acid o-methyltransferase; CCoAOMT, caffeoyl-CoA o-methyltransferase; F5H, ferulate-5-hydroxylase. The flavonoids biosynthetic branch: CHS, chalcone synthase; CHI, chalcone isomerase; F3H, flavanone 3-hydroxylase; F3′H, flavonoid 3′-hydroxylase; FLS, flavonol synthase. Dash arrows refer to unspecified steps of a particular metabolic pathway. The colored boxes indicate metabolites that are significantly induced by both types of stress.

Additionally, four flavones were also accumulated in response to both stresses. Flavones are a kind of flavonoid which are also derived from the phenylpropanoid metabolic pathway. All flavonoid compounds are composed of two benzene rings connected by a 3-carbon linking chain (Nabavi et al., 2018). Ring A is synthesized from three malonyl-CoA molecules generated *via* the transformations of glucose while ring B is synthesized from 4-coumaroyl-CoA produced from phenylalanine (Figure 8). Rings A and B condense to generate chalcone, and then transform to flavanone via isomerase-catalyzed cyclization. Flavanone is the starting compound for the synthesis of other flavonoids (Nabavi et al., 2018). Flavones are converted from flavanones through flavone synthase. Flavones, flavonols, and anthocyanins accumulating in leaf epidermal cells, waxes, and trichomes can act as UV-B filters and form DNA crosslinking to protect DNA from oxidative damage (Dixon, 2005; Aron and Kennedy, 2008; Albert et al., 2009; Hichri et al., 2011). In plateau environments, the high UV and harsh abiotic stresses probably cause over-production of ROS. The four flavones are likely to be involved in anti-oxidation protection of plants. Thus, it can be seen that phenylpropanoid metabolism is a vital reason why *Poa* acquired multi-tolerance in our study.

**Figure 9:**
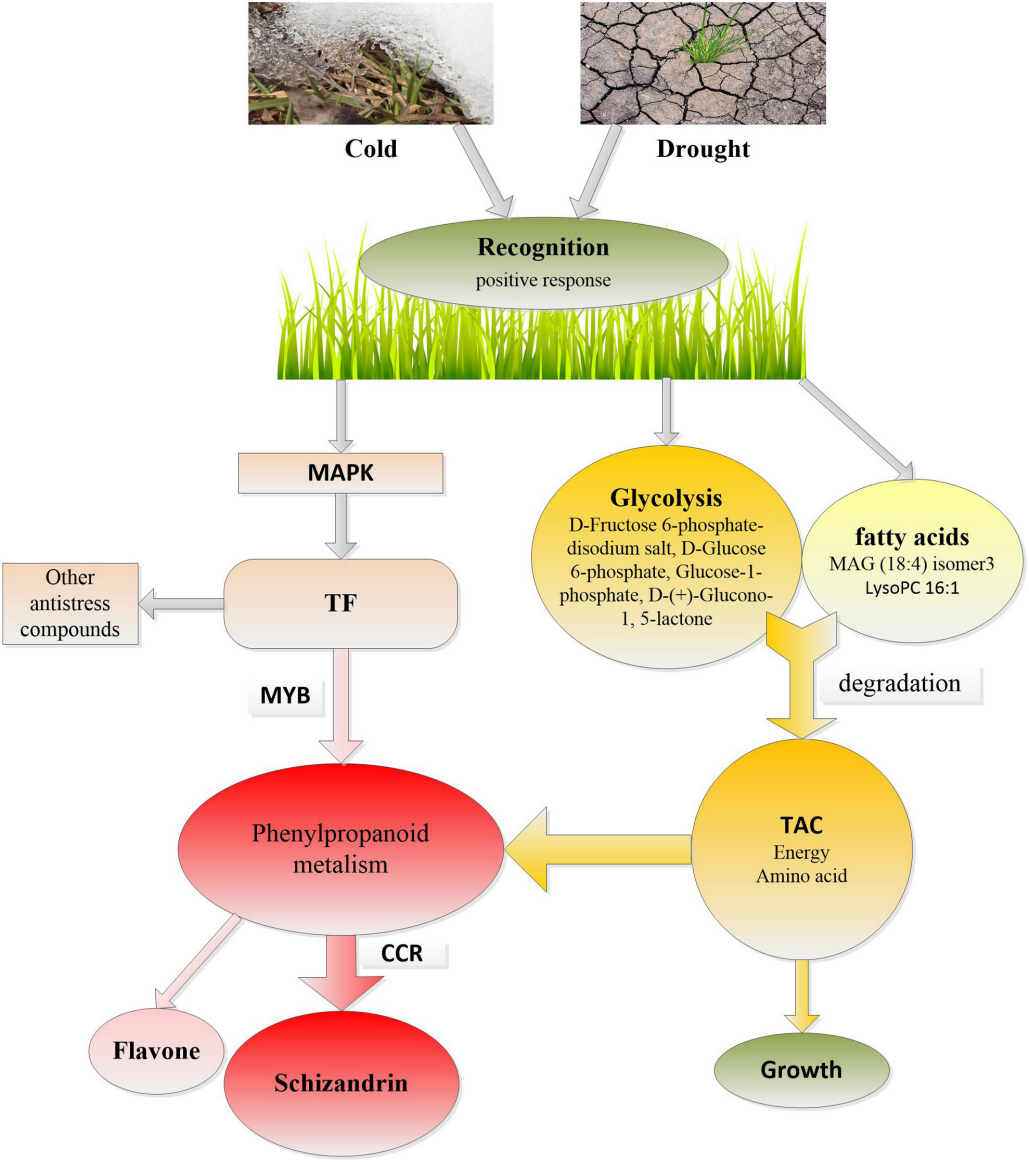
The response of *Poa crymophila Keng* to cold and drought stresses. TAC, tricarboxylic acid cycle; CCR, cinnamoyl-CoA reductase; TF, transcription factor; MAPK, mitogen-activated protein kinase.

In addition, four carbohydrates, such as D-Fructose 6-phosphate-disodium salt, D-Glucose 6-phosphate, Glucose-1-phosphate, and D-(+)-Glucono-1,5-lactone, which are intermediate products in the glycolysis pathway and two unsaturated lipids, were amongst the 17 significantly induced metabolites (Figure 2). Glycolysis and FA degradation participating in the tricarboxylic acid cycle is the main source of amino acids, which is inseparable from the synthesis of metabolites in the phenylpropanoids pathway (Figure 9).

### The Correlated Genes and Their Pathways

The genes which were positively correlated with the 17 metabolites (PCC >0.9) were identified and filtered on the basis of the gene length exceeding 750 bp. Because the genes correlated with each metabolite overlapped, we analyzed them together by mapping them in Mapman to reveal their main pathways. Sucrose-starch and raffinose metabolism were the chief pathways in primary metabolism, while the phenlypropanoid pathway functioned most notably in secondary metabolism (Figures 3, 4). According to the expression level of each gene and the expression differences between the two groups, the key genes were identified.

In primary metabolism, the key genes were mainly alpha-amylase, beta-fructofuranosidase, raffinose synthase, and sugar porter family MFS transporter (Table 3). Glucose and starch were degraded to accelerated glycolysis to cope with drought and cold stress (Bhargava and Sawant, 2013, Morkunas and Ratajczak, 2014). The status and allocation of carbohydrates enabled more energy to be available for dealing with environmental stresses rather than for growth. This may also explain why plants on the plateau tend to be dwarfed.

In secondary metabolism, CCR (EC 1.2.1.44) showed significant up-regulation in plants in response to both stresses. CCR catalyzed the first reaction of monolignol synthesis in a pivotal position of the phenylpropanoid pathway and directed metabolic flux toward a different direction of monolignols or flavonoids (Sattler et al., 2017). In *Poa*, significantly induced CCR and the substantial enrichment of schizandrin indicated that metabolic flux was more oriented to monolignols synthesis (Figure 8).

On the other hand, the remainder of the metabolic flux in the phenylpropanoid pathway flowed into the biosynthesis of flavones (Figure 8). In dicots, O-glycosylated flavonols are the major type of flavonoids, while monocot species predominantly produce flavone C-glycosides, including chrysin, apigenin, luteolin, and tricin, which have been detected in wheat, rice, and maize (Tohge et al., 2017). In *Poa*, four flavones, including luteolin, tricin, and apigenin, were significantly upregulated under both stresses (Figure 2), which suggested that *Poa* accords with monocot species. However, we only detected flavonol synthase (FLS) and did not find flavone synthase in the *Poa* transcriptome. There may be other pathways to induce flavone synthesis.

Many transcription factors, such as MYB, WRKY, and NAC, can regulate the synthesis of many compounds in phenylpropanoid pathways (Gray et al., 2012; Mierziak et al., 2014; Nabavi et al., 2018). In the 4,286 associated genes (>750 bp), there were nine MYB, three WEKY, and one NAC identified in our study, and only one MYB was remarkably upregulated by both cold and drought stresses. The direct targets of many of these TFs remain unknown, and cross regulation between TFs may also exist. Although it has been proved that biotic and abiotic stresses can trigger lignin and flavonoids in many plants (Gray et al., 2012), the factors and pathways of regulation require further study. In addition, in the data concerning all DEGs and the correlated genes, there were more unigenes in response to drought than cold. Thus, we inferred that drought is probably the main stress factor affecting the survival of *Poa* in cold and arid areas.

## Conclusion

In this study, we focused on *P. crymophila Keng*, an excellent forage grass, and identified 779 metabolite features and 167,845 unigenes. There were 17 metabolites which were significantly induced by both stresses, mainly carbohydrates, flavones, and phenylpropanoids. Among them, schizandrin (pmf0166), a kind of lignan, likely has the closest connection to the tolerance of the plant because it showed the highest fold change (over 10,000 times). A total of 4,286 upregulated genes (>750 bp) were positively related to the 17 metabolites with PCC >0.9. The key genes included alpha-amylase, beta-fructofuranosidase, and genes related to sugar transport in primary metabolism; and cinnamoyl-CoA reductase, flavonol synthase, and MYB in secondary metabolism. Glucose and starch were degraded to small molecule sugars to support the growth of *P. crymophila* under adverse environmental conditions. Phenylpropanoid metabolism appears to be a vital reason why *Poa* has acquired multi-tolerance capabilities because of the accumulation of schizandrin and flavones in phenylpropanoid pathways. This study presented the mechanism of Poa adapting to multi-stresses and provided a new anti-stress substance that can be used to improve the tolerance of crops in adverse environments.

## Data Availability Statement

The datasets presented in this study can be found in online repositories. The names of the repository/repositories and accession number(s) can be found in the article/Supplementary Material.

## Author Contributions

YW and X-RM designed the experiment. YW completed the data analysis and the manuscript writing. X-YL participated in analyzing the sequence data. C-XL, YH, and X-YH participated in preparing, treating, and collecting samples. X-RM revised the manuscript. All authors read and approved the final manuscript.

## Conflict of Interest

The authors declare that the research was conducted in the absence of any commercial or financial relationships that could be construed as a potential conflict of interest.

## Supplementary Material

Supplementary Table 1 Gene expressions of *Poa crymophila Keng* cv. Qinghai transcriptome and gene expression differences in response to cold and drought stresses.

Supplementary Table 2 Annotation of all unigenes in *Poa crymophila Keng* cv. Qinghai transcriptome.

Supplementary Table 3 *Poa crymophila Keng* cv. Qinghai metabolome data in response to cold and drought.

Supplementary Table 3-S1 Information on all metabolites in *Poa crymophila Keng* cv. Qinghai metabolome.

Supplementary Table 3-S2 Significantly differentially expressed metabolites in Cold-vs.-CK.

Supplementary Table 3-S3 Significantly differentially expressed metabolites in Drought-vs.-CK.

Supplementary Table 3-S4 Significantly differentially expressed metabolites in ReCold-vs.-CK.

Supplementary Table 3-S5 Significantly differentially expressed metabolites in ReDrought-vs.-CK.

Supplementary Table 3-S6 Significantly differentially expressed metabolites in ReCold-vs.-Cold.

Supplementary Table 3-S7 Significantly differentially expressed metabolites in ReDrought-vs.-Drought.

Supplementary Table 3-S8 Significantly upregulated metabolites in both cold and drought stresses.

Supplementary Table 4 Correlation analysis between induced metabolites under two stresses and upregulated genes.

Supplementary Table 4-S1 The length of each gene in the *Poa crymophila Keng* cv. Qinghai transcriptome.

Supplementary Table 4-S2 Genes that are positively related to the 17 metabolites with Pearson's correlation coefficients (PCC) >0.9. Genes with length exceeding 750 bp are highlighted in yellow.

Supplementary Table 4-S3 Genes mapped in secondary metabolism using MCC and Mapman software.

Supplementary Table 4-S4 Genes mapped in primary metabolism using MCC and Mapman software.
